# An Accurate Kinematic Analysis with Clinical Convenience for Decomposing Mandibular Movement into Translational and Rotational Components: A Preliminary Proof-of-Concept Study

**DOI:** 10.3390/bioengineering13060645

**Published:** 2026-05-29

**Authors:** Youyi He, Baotian Zhu, Haolin Li, Deqiang Yin, Yang Liu

**Affiliations:** 1 College of Aerospace Engineering, Chongqing University, No. 174 Shazhengjie, Shapingba District, Chongqing 400044, China; youyihe@stu.cqu.edu.cn (Y.H.); zhubaotian0424@163.com (B.Z.); 2 State Key Laboratory of Oral Disease, National Clinical Research Center for Oral Disease, West China Hospital of Stomatology, Sichuan University, No. 24, Yihuan Road, Wuhou District, Chengdu 610065, China; lihl2021@163.com

**Keywords:** decomposing movement, kinematic parameters, bite-guiding splint, finite helical axis (FHA)

## Abstract

Motion dysfunction constitutes a critical clinical manifestation of temporomandibular joint disorder (TMD). Notably, prior research predominantly consists of qualitative clinical descriptions. To better understand the mandibular movement by quantitative analysis, our study proposes a mathematical approach for extracting kinematic parameters and preliminarily illustrates the feasibility and technical advantages through a comparative kinematic parameters analysis of a volunteer opening–closing movement performed in maximum intercuspal position (MIP) and therapeutic position (TP). By decomposing the translational and rotational components of opening–closing movement, the symmetry of the mandibular movement was improved and the maximum mouth opening was increased after wearing a bite-guiding splint. Furthermore, changes in the spatial position of the finite helical axis (FHA) indicated that the FHA position became more stable after wearing the splint, suggesting that this metric has potential kinematic assessment value. Finally, a cushioning effect was observed at the maximum mouth opening when the bite-guiding splint was worn. This approach is expected to provide clinical practice with a high-precision digital assessment tool and a valuable reference for the quantitative evaluation of personalized temporomandibular joint (TMJ) functional rehabilitation. The method could not only conveniently extract the key kinematic parameters of mandible movement but also efficiently evaluate the therapeutic outcomes of clinical intervention.

## 1. Introduction

Mandible kinematic characteristics are pivotal for evaluating the functionality of the temporomandibular joint (TMJ), which involves quantifying parameters such as joint movement trajectory, ranges, and velocity of the joint structure. In clinical treatment, mandibular movement changes are commonly used to assess joint function. Patients with intra-articular structural disorders typically exhibit a restricted range of motion, non-smooth movement, and joint clicking [1]. However, current evaluation methods mainly depend on doctors’ subjective judgment, lacking precise quantitative indicators. The abnormal mandibular movement is frequently associated with pathologies pertaining to the TMJ, which is most prevalent in temporomandibular joint disorder (TMD) [2,3,4]. This condition encompasses a spectrum of structural and functional disorders that, in turn, affect the joint and its associated oral muscles, often leading to compromised mandibular movement and a range of clinical manifestations [5,6]. Consequently, implementing a quantitative description of mandibular movement is essential to elucidate TMD pathogenesis, optimize individualized therapeutic strategies, and objectively assess rehabilitation outcomes [7,8].

Mandibular movement in space is exceedingly complex, roughly involving both translational and rotational components, which could be characterized as an intricate three-dimensional (3D) motion [9,10]. Clinically, digital articulators are used to simulate the movements of the mandible relative to the skull through hinges and guide rails. However, relatively rigid structures are unable to accurately reproduce physiological joint movement primarily due to both insufficient degrees of freedom (DOFs) and a lack of individualized adaptability [11]; the key to resolving this issue lies in the precise quantitative characterization of mandibular movement. The history of mandibular movement recording technology can be broadly divided into three stages. Initially, it mainly relied on clinicians’ visual observation and manual tracing [12]. Since the 20th century, various mechanical devices have enabled quantitative tracking of mandibular movements [13,14]. With the development of modern optical and electromagnetic technologies, mandibular movement recording has entered an era of electronic and digital technology [15,16]. Although optical [17,18] and electromagnetic [19,20] tracking technologies have enabled the multifaceted acquisition of mandibular movements, many related studies have been limited to qualitative descriptions of discrete points of mandibular movements [21,22,23], and there is lack of insightful quantitative research on the implications of changes in intrinsic kinematic parameters, such as rotational velocity and the position of finite helical axis (FHA) [24,25,26].

To address the limitations of qualitative trajectory descriptions, this study proposes a rigid-body kinematics framework to extract intrinsic parameters from 3D discrete point trajectories. This approach would exhibit higher sensitivity in capturing kinematic differences before and after clinical interventions than traditional trajectory analysis. As a preliminary proof-of-concept, we performed a comparative kinematic analysis of a volunteer opening–closing movement in both maximum intercuspal position (MIP) and therapeutic position (TP) with a bite-guiding splint. This approach is expected to provide clinical practice with a non-invasive and high-precision digital assessment tool. Once validated in future studies with larger sample sizes, it could serve as a valuable reference for the quantitative evaluation of personalized TMJ functional rehabilitation.

## 2. Materials and Methods

### 2.1. Data Collection

A 40-year-old male volunteer met the following criteria: (1) complete permanent dentition with stable occlusal points; (2) no abnormal oral or maxillofacial disorders; (3) no history of TMD. The volunteer signed informed consent for the use of his collected imaging data for scientific research and educational activities.

**Figure 1:**
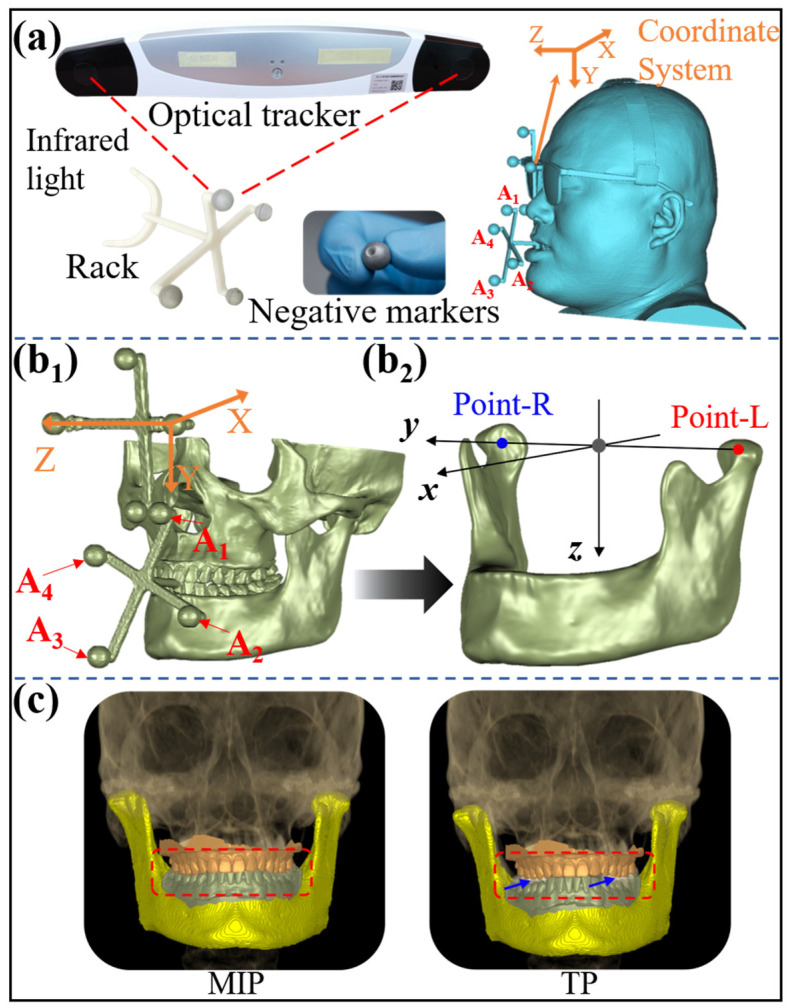
(**a**) Schematic diagram of the NDI Polaris Spectra acquisition instrument. (**b**) The transformation from (**b_1_**) the NDI data collection coordinate system (X, Y, Z) to (**b_2_**) the clinical coordinate system (*x*, *y*, *z*). (**c**) The different initial jaw positions in MIP and TP, respectively.

The NDI Polaris Spectra (Polaris Vega System version, Northern Digital Inc., Waterloo, ON, Canada) passive optical motion tracking system was used to capture mandibular movement at 50 Hz, which determines 3D marker coordinates via infrared-based triangulation. According to NDI design guidelines, two rigid navigation racks with four markers each were 3D-printed using lightweight plastic materials instead of steel to minimize movement interference. Each rack features a rigid structure with four fixed markers to ensure no relative movement among the markers. To ensure rigid coupling, the maxillary rack was securely fixed to the upper jaw by using a clamp, while another rack with four markers, labeled as *A*_1_–*A*_4_, was bonded directly to the lower jaw teeth. Before data collection, CT scans were performed with the racks in place to align the markers with the mandibular coordinate system, minimizing errors arising from coordinate system registration. Specifically, a sufficient number of points (more than 1000 points) distributed across the entire surface of each marker were manually selected to fit the sphere’s geometric centers. These fitted centers were registered with the marker coordinates recorded by the NDI system. Subsequently, under the guidance of an experienced dentist, the volunteer performed a standardized opening–closing movement, as shown in Figure 1a.

As illustrated in Figure 1b_1_, the NDI system tracks four mandibular markers ($A_{1}–A_{4}$) relative to the maxillary reference rack. The native coordinate system (X, Y, Z) is defined with the *Z*-axis extending leftward, the *Y*-axis downward, and the *X*-axis inward. To align with clinical practice, the native coordinate system (X, Y, Z) was transformed into a standardized clinical coordinate system (*x*, *y*, *z*) (Figure 1b_2_). The initial position was set at the MIP. The *y*-axis is defined as the line connecting the geometric centers of the left and right condyles, the *x*-axis follows the anteroposterior direction, and the *z*-axis extends vertically downward. This clinical coordinate system adheres to the right-hand Cartesian rule.

To more clearly quantify mandibular movement across different conditions, this study collected data from a volunteer who performed five repetitions of opening–closing movements at two different initial jaw positions (Figure 1c). Condition I represented the MIP without clinical intervention. Meanwhile, Condition II represented the TP by using a bite-guiding splint to simulate clinical intervention. Under the guidance of an experienced clinician, the volunteer maintained a physiologically relaxed state before data collection. Medical imaging provided clear evidence that the bite-guiding splint alters the initial occlusal jaw position, where the red dashed box highlights the splint position.

### 2.2. Reconstruction of the Kinematic Model

The positional relationship for any point *A_i_*(*x_i_*,*y_i_*,*z_i_*) in a rigid body between consecutive time intervals (Δ*t*, $t_{i}\overset{∆t}{\to }t_{i+1}$) can be defined by using a rotation matrix $R^{t_{i}}$ and a translation matrix $T^{t_{i}}$:

(1) $$A_{i}^{t_{i+1}}\left(x_{i}^{t_{i+1}},y_{i}^{t_{i+1}},z_{i}^{t_{i+1}}\right)=R^{t_{i}}A_{i}^{t_{i}}\left(x_{i}^{t_{i}},y_{i}^{t_{i}},z_{i}^{t_{i}}\right)+T^{t_{i}}.$$

In our study, the four markers ($A_{1}–A_{4}$) on the navigation rack were coplanar, which makes it difficult to determine ***R*** and ***T*** by using a homogeneous transformation matrix (Supplementary Materials S1). Mathematically, a plane defined by three non-collinear points is sufficient to represent the six DOFs of a rigid body. Consequently, ***R*** and ***T*** are calculated by using three non-collinear points. To validate the feasibility and high precision of the proposed method, the coordinates of the fourth point (*A*_4_) are reconstructed and compared against its actual trajectory. At a given moment $t_{i}$, the spatial positions of the first three points are denoted as: $A_{1}^{t_{i}}\left(x_{1}^{t_{i}},y_{1}^{t_{i}},z_{1}^{t_{i}}\right)$, $A_{2}^{t_{i}}\left(x_{2}^{t_{i}},y_{2}^{t_{i}},z_{2}^{t_{i}}\right)$ and $A_{3}^{t_{i}}\left(x_{3}^{t_{i}},y_{3}^{t_{i}},z_{3}^{t_{i}}\right)$, wherein the superscript ($t_{i}$) indicates the moment $t_{i}$ and the subscript (1, 2, 3) indicates the numbered points. Then, an instantaneous plane at moment $t_{i}$ could be uniquely determined by th following three points:

(2) $$a^{t_{i}}x+b^{t_{i}}y+c^{t_{i}}z+d^{t_{i}}=0.$$

The coefficients (${a^{t_{i}},{\;b}^{t_{i}},{\;c}^{t_{i}},\;d}^{t_{i}}$) of Equation (2) can be calculated as follows:

(3) $$a^{t_{i}}=\left(y_{2}^{t_{i}}-y_{1}^{t_{i}}\right)\left(z_{3}^{t_{i}}-z_{1}^{t_{i}}\right)-\left(y_{3}^{t_{i}}-y_{1}^{t_{i}}\right)\left(z_{2}^{t_{i}}-z_{1}^{t_{i}}\right),$$

(4) $$b^{t_{i}}=\left(z_{2}^{t_{i}}-z_{1}^{t_{i}}\right)\left(x_{3}^{t_{i}}-x_{1}^{t_{i}}\right)-\left(z_{3}^{t_{i}}-z_{1}^{t_{i}}\right)\left(x_{2}^{t_{i}}-x_{1}^{t_{i}}\right),$$

(5) $$c^{t_{i}}=\left(x_{2}^{t_{i}}-x_{1}^{t_{i}}\right)\left(y_{3}^{t_{i}}-y_{1}^{t_{i}}\right)-\left(x_{3}^{t_{i}}-x_{1}^{t_{i}}\right)\left(y_{2}^{t_{i}}-y_{1}^{t_{i}}\right),$$

(6) $$d^{t_{i}}=-a^{t_{i}}x_{1}^{t_{i}}-b^{t_{i}}y_{1}^{t_{i}}-c^{t_{i}}z_{1}^{t_{i}}.$$

The coordinate matrix at moment $t_{i}$, is denoted as $M^{t_{i}}{:(A_{1}^{t_{i}},{\;A}_{2}^{t_{i}},{\;A}_{3}^{t_{i}})}^{T}$. Similarly, the coordinate matrix at time $t_{i+1}$ is denoted as $M^{t_{i+1}}:{\left(A_{1}^{t_{i+1}},A_{2}^{t_{i+1}},A_{3}^{t_{i}}\right)}^{T}$. The transformation matrix ($P^{t_{i}}$) from $M^{t_{i}}$ to $M^{t_{i+1}}$ can be expressed as Equation (7):

(7) $$P^{t_{i}}=M^{t_{i+1}}{{(M}^{t_{i}})}^{-1}.$$

Define Equations (8)–(10) to determine the transformation matrix, $R^{t_{i}}$ and $T^{t_{i}}$.

(8) $$P^{t_{i}}=\left(\begin{array}{ccc} P_{11} & P_{12} & P_{13}\\ P_{21} & P_{22} & P_{23}\\ P_{31} & P_{32} & P_{33} \end{array}\right),$$

(9) $$R^{t_{i}}=\left(\begin{array}{ccc} R_{11} & R_{12} & R_{13}\\ R_{21} & R_{22} & R_{23}\\ R_{31} & R_{32} & R_{33} \end{array}\right),$$

(10) $$T^{t_{i}}=\left(\begin{array}{c} T_{1}\\ T_{2}\\ T_{3} \end{array}\right).$$

According to Equation (2), the points $N^{t_{i}}(x,y,z$) on the plane can be expressed as follows:

(11) $${\left(x,\;y,\;-\frac{d+ax+by}{c}\right)}^{T}.$$

Since the motion on the plane within 3D space is also in a 3D form, possessing six DOFs, it can be described using a rotation matrix ($R^{t_{i}}$) and a translation matrix ($T^{t_{i}}$). Therefore, Equation (12) could be established:

(12) $$P^{t_{i}}N^{t_{i}}=R^{t_{i}}N^{t_{i}}+T^{t_{i}}.$$

Satisfying Equations (13)–(27):

(13) $$P_{11}-P_{13}a/c=R_{11}-R_{13}a/c.$$

(14) $$P_{12}-P_{13}b/c=R_{12}-R_{13}b/c.$$

(15) $$P_{13}d/c=R_{13}d/c+T_{1}.$$

(16) $$P_{21}-P_{23}a/c=R_{21}-R_{23}a/c.$$

(17) $$P_{22}-P_{23}b/c=R_{22}-R_{23}b/c.$$

(18) $$P_{23}d/c=R_{23}d/c+T_{2}.$$

(19) $$P_{31}-P_{33}a/c=R_{31}-R_{33}a/c.$$

(20) $$P_{32}-P_{33}b/c=R_{32}-R_{33}b/c.$$

(21) $$P_{33}d/c=R_{33}d/c+T_{3}.$$

(22) $$R_{11}R_{11}+R_{12}R_{12}+R_{13}R_{13}=1.$$

(23) $$R_{21}R_{21}+R_{22}R_{22}+R_{23}R_{23}=1.$$

(24) $$R_{31}R_{31}+R_{32}R_{32}+R_{33}R_{33}=1.$$

(25) $$R_{11}R_{12}+R_{21}R_{22}+R_{31}R_{32}=0.$$

(26) $$R_{11}R_{13}+R_{21}R_{23}+R_{31}R_{33}=0.$$

(27) $$R_{12}R_{13}+R_{21}R_{23}+R_{31}R_{33}=0.$$

Solving the equational groups with twelve unknown constants (nine rotational components and three translational components) based on the above fifteen equations (Equations (13)–(27)), a determinant could be built to uniquely determine the existing $R^{t_{i}}$ and $T^{t_{i}}$. Since the rigid body will not be scaled or contracted during the rotation process, $R^{t_{i}}$ should satisfy

(28) $$Det\left(R^{t_{i}}\right)=1.$$

The rotation angle ($\theta^{t_{i}}$) and rotation axis vector ($\overset{⃑}{n}$) at the adjacent time can also be given by the Rodrigues’ formula, as shown in Equations (29) and (30):

(29) $$\theta^{t_{i}}=arccos\left[\left(tr\left(R^{t_{i}}\right)-1\right)/2\right].$$

(30) $$R^{t_{i}}\overset{⃑}{n}=\overset{⃑}{n},\;\overset{⃑}{n}=(n_{x},n_{y},n_{z}{)}^{T}.$$

### 2.3. The Determination of Finite Helical Axis (FHA)

While $R^{t_{i}}$ and $T^{t_{i}}$ describe rigid body movement from moment *t_i_* to *t_i_*_+1_, they imply a sequential dependency, either rotation preceding translation (Equation (1)) or vice versa (Equation (31)).

(31) $$A_{i}^{t_{i+1}}\left(x_{i}^{t_{i+1}},y_{i}^{t_{i+1}},z_{i}^{t_{i+1}}\right)=R^{t_{i}}\left(A_{i}^{t_{i}}\left(x_{i}^{t_{i}},y_{i}^{t_{i}},z_{i}^{t_{i}}\right)+T^{t_{i}}\right).$$

**Figure 2:**
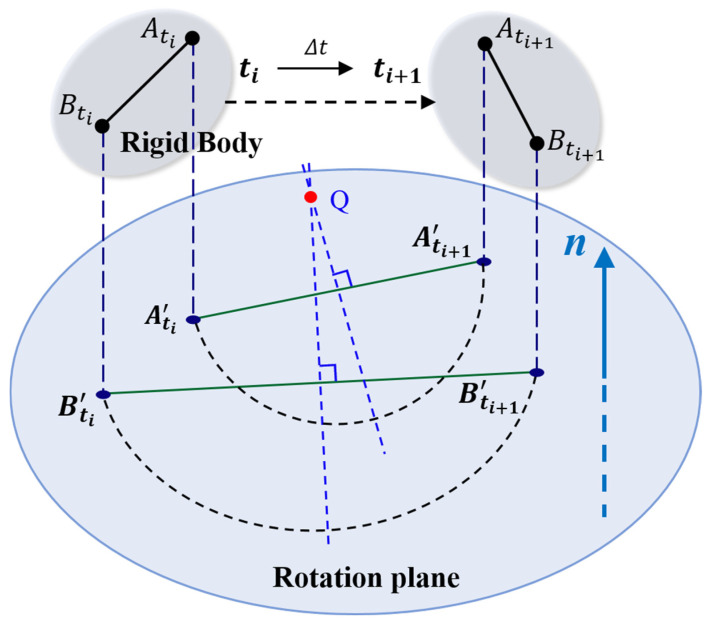
The schematic plot for determining the rotation center (Q) via separating rotational motion and translational motion.

However, physical motion involves the simultaneity of translation and rotation. According to Screw Theory, the motion can be decomposed into two components: a rotation about and a translation along the finite helical axis (FHA) (Supplementary Materials S2). As shown in Figure 2, while ***R*** defines the rotation axis vector ($\overset{⃑}{n}$), the spatial position of the FHA requires identifying a point Q on the axis. At moment *t_i_*, there are two points $A_{t_{i}}$ and $B_{t_{i}}$ on the rigid body, moved to $A_{t_{i+1}}$ and $B_{t_{i+1}}$ at moment *t_i_*_+1_. Projecting the trajectories onto the rotation plane (orthogonal to $\overset{⃑}{n}$) reduces the motion to a pure circular rotation. The rotation center Q, determined by the intersection of the perpendicular bisectors of the segments connecting corresponding projected points, lies on the FHA. Consequently, the FHA is uniquely defined in 3D space by the vector $\overset{⃑}{n}$ and point Q.

### 2.4. The Pre-Process of Collected Data

The NDI system recorded raw trajectories of markers *A*_1_–*A*_4_ at both MIP and TP (Figure S1, Supplementary Materials S3). These trajectories distinctly revealed that TP groups consistently achieved a higher degree of overlap in opening–closing movement compared to the MIP groups. According to the NDI guidelines, the more markers on the navigation rack, the larger the rack becomes, resulting in increased weight and volume, which in turn may interfere with the volunteer’s mandibular movement. Compared to other similar studies [27,28,29], our method requires a minimum of only three markers to infer the entire mandibular movement, possibly reducing the interference in volunteer mandibular movement. In the current study, we totally used four markers wherein the first three markers (*A*_1_–*A*_3_) were adopted for deriving the motion matrix ($R^{t_{i}}$ and $T^{t_{i}}$) and the fourth one (*A_4_*) was utilized for validating the effectiveness of the proposed method by comparing the derived trajectory (DeT) of the fourth point with the actual captured trajectory (CaT).

**Figure 3:**
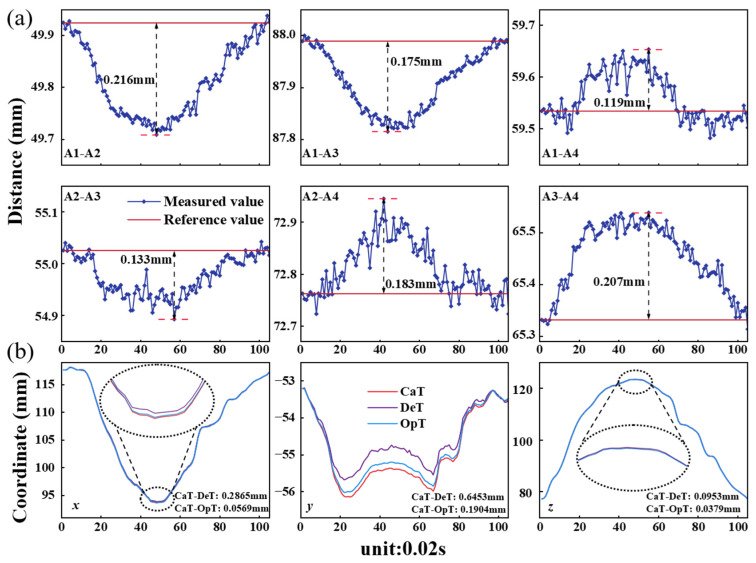
(**a**) The distance change among four markers during opening–closing movement. (**b**) The comparison of the three coordinate axes orientations among the captured trajectory (CaT) (red line), derived trajectory (DeT) (purple line)and optimized trajectory (OpT) (blue line).

Although the NDI Polaris Spectra instrument has been reported to be a high-precision, high-frequency optical tracking device, deriving the motion matrices (***R*** and ***T***) from three markers requires the rigid and invariant distances among the three points. It is difficult to meet this requirement due to the captured error of NDI. To illustrate this point, we selected a set of data collected during mouth opening–closing movement, and calculated the mutual distances (Figure 3a) between the points at each time point using Equation (32):

(32) $$d=\sqrt{(x_{i}-x_{j}{)}^{2}+(y_{i}-y_{j}{)}^{2}+(z_{i}-z_{j}{)}^{2}}.$$

where $A_{i}\;(x_{i},y_{i},z_{i})$ and $A_{j}\;(x_{j},y_{j},z_{j})$ are two coordinate values of markers (i, j= 1, 2, 3, 4, and i≠j). The initial distances (at t = 0) among the first three ones are represented as red horizontal lines in Figure 3a. As one can see, the mutual distance between any two points fluctuates within a small range during opening–closing movement, although this difference is small. The maximum deviation of 0.216 mm for the distances of any two points in the current dataset occurred in $A_{1}–A_{2}$ and falls within the accuracy range reported in the NDI tool design guidelines. However, the deviation remains a significant challenge for deriving a high-precision motion matrix that accurately reflects real mandibular movement when only three points are used. To meet the requirements for deriving a high-precision motion matrix, the raw data is proposed to be preprocessed through quadratic sequence optimization, wherein the initial distances (at t = 0, red horizontal lines) among any two points are adopted as optimization constraints, with the constraint function Equation (33):

(33) $$\frac{{\left\|A_{i}-A_{j}\right\|}^{2}-d_{ij}^{2}}{d_{ij}^{2}}=0.$$

where $A_{i}\;(x_{i},y_{i},z_{i})$ and $A_{j}\;(x_{j},y_{j},z_{j})$ are two coordinate values of markers i and j, $d_{ij}$ is the referenced value between markers i and j (i,j= 1, 2, 3, 4, and i≠j).

As shown in Figure 3a, the distances are consistent at the starting and ending positions of the movement, suggesting that the data collected in the stationary state is relatively accurate. However, additional errors may arise during motion due to lighting conditions, the lag between real-time motion and infrared light feedback, and the instrument’s algorithm, etc. By calculating the gradient of the constraint condition for each pair of points, we can construct the gradient matrix for the entire set of pairs. The larger the gradient of the constraint function for a pair of points, the greater the deviation between the distance of that pair and the referenced value, and the greater the adjustment required for the points within that pair. By constructing the gradient matrix for the entire point pair data, we consider dynamic adjustments for each point across the entire domain, ensuring that the preprocessed data achieves the optimal solution. The entire optimization process is implemented in MATLAB (version 2014a) by using customized codes. As mentioned earlier, the fourth point (*A*_4_) can be used to evaluate trajectory error. Here, we selected the MIP initial jaw position data of point *A*_4_ to demonstrate the effectiveness of data optimization.

As shown in Figure 3b, the data is projected onto the x, y, and z coordinate axes. The data captured by the NDI instrument at point *A*_4_ is represented by a red line, defined as the captured trajectory (CaT); the trajectory of point *A*_4_ directly calculated from the first three points *A*_1_, *A*_2_, and *A*_3_ is represented by a purple line, defined as the derived trajectory (DeT); the data of point *A*_4_ after optimization is represented by a blue line, defined as optimized trajectory (OpT).

The maximum deviations between every two trajectories were summarized in Table 1. Before optimization, the maximum differences between the DeT and the CaT in the three components were x: 0.2856 mm, y: 0.6453 mm, and z: 0.09 mm. After optimization, the maximum differences between the OpT and the CaT in the three components were x: 0.0569 mm, y: 0.1904 mm, and z: 0.0379 mm. From the geometric shapes of the trajectories in the three directions, the DeT and CaT showed consistency, indicating that the method of solving the motion matrix by using three non-collinear points is effective. On the other hand, the OpT was closer to the CaT compared to the DeT, suggesting that the quadratic sequence optimization method we adopted is a satisfactory and effective optimization method.

## 3. Results

### 3.1. Translational Movement

Based on the decomposition and accumulation of translational displacements between consecutive frames in Equation (34):

(34) $$x=\sum_{start}^{end}x_{i},\;y=\sum_{start}^{end}y_{i},\;z=\sum_{start}^{end}z_{i}.$$

**Figure 4:**
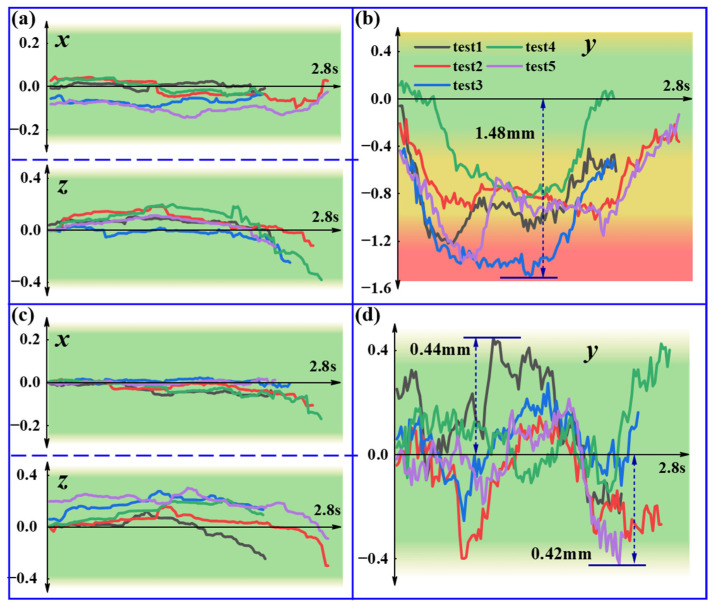
Volunteer translation increment during opening–closing movement at two different initial jaw positions. MIP groups: (**a**) x, z components; (**b**) y components. TP groups: (**c**) x, z components; (**d**) y component. There are five groups of test data.

The color backgrounds employed in Figure 4 aim to intuitively represent the degree of translational offset. The green background represents the healthy range (0–0.4 mm). The yellow background represents the warning range (0.4–0.8 mm). The red background represents the high-risk range (over 0.8 mm). As shown in Figure 4, both in the MIP and TP groups, the translation in the x-component approached zero. Although in the TP groups, translation in the z-component shifted slightly further downward, the overall displacement remained highly limited, with the maximum deviation from the balance position less than 0.4 mm. Contrastingly, a significant difference can be observed in the y-component. In MIP groups, the mandible shifted laterally and returned to the center during closing. Conversely, in TP groups, the y-component was maintained within a low range dynamic equilibrium without unilateral shifting. It is noted that translational velocity was excluded from this analysis, as the minimal displacement is highly susceptible to measurement noise and distortion at a high frequency.

### 3.2. Rotational Movement

The projected and accumulated rotational angle increments, based on Equation (35), are as follows:

(35) $$\theta_{x}=\sum_{start}^{end}\theta_{x_{i}},\;\theta_{y}=\sum_{start}^{end}\theta_{y_{i}},\;\theta_{z}=\sum_{start}^{end}\theta_{z_{i}}.$$

**Figure 5:**
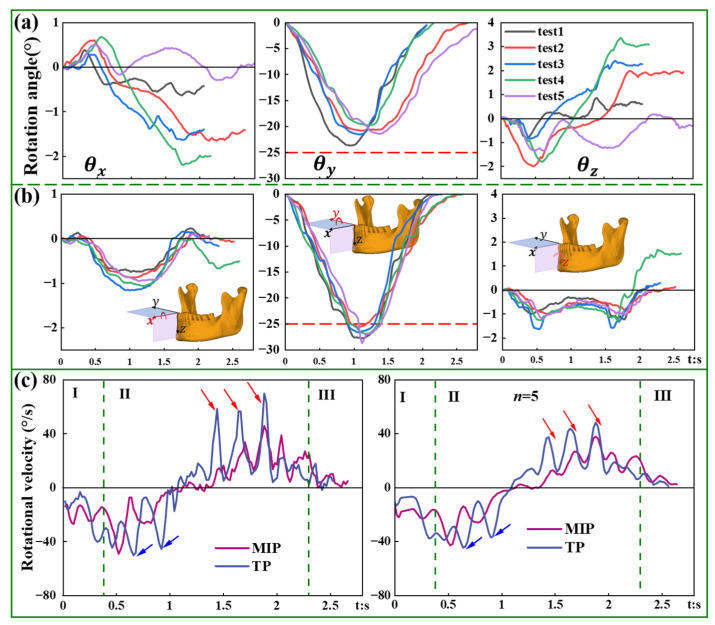
Rotation angle increment (θ) during opening–closing movement at two different initial jaw positions. (**a**): MIP groups; (**b**): TP groups. (**c**): The angular velocity (ω) variation for before homogeneity and after homogeneity (*n* = 5).

As shown in Figure 5a,b, in the MIP groups, the rotation angles around the *x*-axis (x-component, $\theta_{x}$) and *z*-axis (z-component, $\theta_{z}$) were larger and more disordered. In the TP groups, these rotation angles were minimized, indicating a significant improvement in rotational symmetry. The maximum rotation angle around the *y*-axis (y-component, $\theta_{y}$) increased from an average of −21.40° in the MIP groups to −27.13° in the TP groups. These findings suggested that the symmetry of the mandibular movement was improved and the maximum mouth opening was increased after the volunteer wore a bite-guiding splint. Since the *y*-axis serves as the primary axis of rotation during opening–closing movement, the rotational velocity ($\omega_{t}$) at each frame around the *y*-axis is further analyzed from Equation (36):

(36) $$\omega_{t}=\frac{d\theta }{dt}.$$

The entire opening–closing movement (0–2.8 s) can be characterized by three stages: (I) initial stage (0.0–0.4 s), where the velocity gradually increases from zero; (II) peak stage (0.4–2.3 s), where the mandible gradually approaches the maximum opening position, reaches it, and then moves away with a relatively steady rotational velocity; (III) closure stage (2.3–2.8 s), where the mandible gradually returns to the initial position with the decreasing rotational velocity. A marked distinction was observed between the MIP and TP groups, as indicated by the blue and red arrows; the TP groups exhibited higher and more sustained peak velocities with fewer fluctuations. Due to the 50 Hz frequency, the raw rotational velocity curves exhibited significant noise. To provide an intuitive visual representation of the alignment variations, a moving-average smoothing filter was applied according to Equation (37):

(37) $$\omega_{i}=\frac{\sum_{j=1}^{\frac{n-1}{2}}\left(w_{i-j}+w_{i-j+1}+\cdots +w_{i}+w_{i+1}+\cdots +w_{i+j}\right)}{n}$$

As shown in Figure 5c, the smoothed profiles (*n* = 5) further reinforce the superior performance of the TP groups, which maintain higher and more consistent rotational velocities than the MIP groups. This trend remains consistent across various smoothing degrees (Figure S2, Supplementary Materials S4), suggesting the bite-guiding splint may have the potential to stabilize opening–closing movement and optimize functional kinetics.

### 3.3. The Position of Finite Helical Axis (FHA)

**Figure 6:**
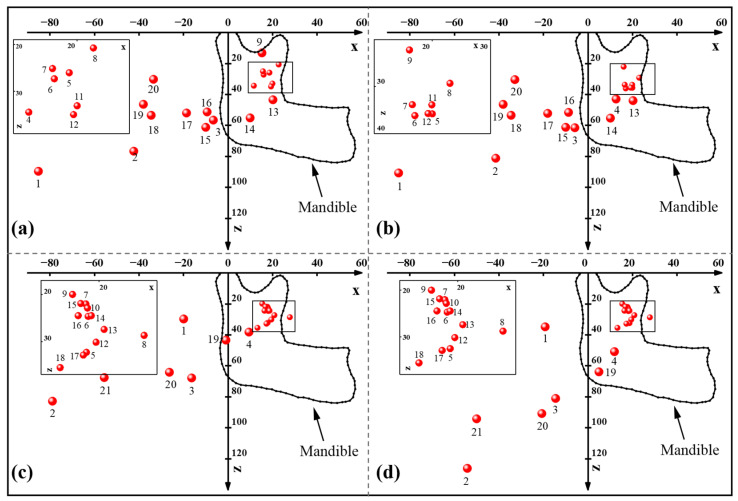
Evolution of FHA: the projected rotation axis on the sagittal plane of (**a**) left and (**b**) right condyles during opening–closing movement for MIP, and (**c**) left, (**d**) right sides for bite-guiding splints (TP). The sequence numbers indicate the progression of the opening–closing movement.

Following the procedure in Section 2.3, the position of FHA with a time interval of 0.1 s was presented in Figure 6. The FHA position demonstrates significant spatial migration, both in the MIP (Figure 6a,b) and TP (Figure 6c,d) groups, exhibiting generally symmetric bilateral distributions, consistent with the fundamental kinematics of mandibular opening–closing movement.

Consistent with the rotational velocity, the position of the FHA follows three functional sequences: (I) initial stage (number 1–4), where the instantaneous FHA is located at a long distant position posterior and inferior to the mandible, the mandible rotates around this rotation axis, while the condyle, being distant from the rotation axis, performs a “translational-like” movement along the condylar slope, sliding forward and inferiorly; (II) peak stage (number 5–14), where the condyle is crossing the condyle tubercle and rotating around it. Since the condyle is now close to the rotation axis, the radius of rotation is small, resulting in rotational movement of the condyle; (III) closure stage (number 15–20), where the condyle moves along the condylar slope, returning to the original position, causing the rotational axis to return to the posterior inferior region of the mandible.

Notably, the initial rotation center (Red Ball 1) in the TP groups was positioned closer to the concentrated region. Moreover, the distribution of projected points for the bite-guiding splint case (TP) is more concentrated during the (II) peak stage, indicating that FHA has the potential to preliminarily assess the resistance to interference during the opening–closing movement.

### 3.4. The Evolution of the Condyle Position Relative to Fossa

**Figure 7:**
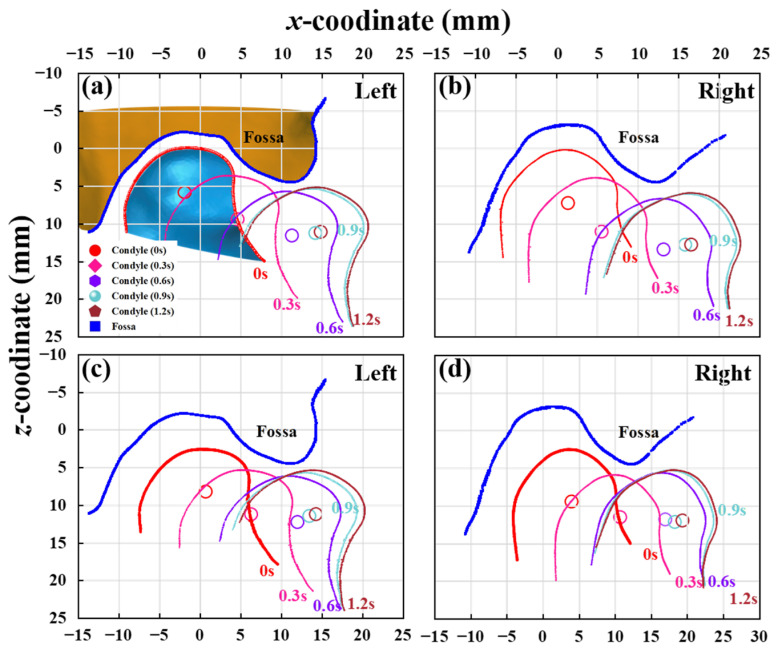
The relative position of the condyle relative to the fossa in the sagittal plane projection during opening movement (0–1.2 s): (**a**) left and (**b**) right sides for MIP; (**c**) left and (**d**) right sides for the TP case (with bite-guiding splint).

With the derivation of ***R*** and ***T***, the relative position of the condyle and fossa at every moment could be reconstructed to explore the relative movement. Figure 7 roughly depicts the relative positions of the condyle and fossa on both sides of the mandible during the opening process (0–1.2 s) in the sagittal plane by using the aggregation of typical points on the boundaries of counterparts.

Compared to the TP groups, the MIP groups exhibited significant non-linear acceleration after crossing the articular tubercle. Specifically, between 0.9 and 1.2 s, the MIP groups demonstrated an impulsive motion with high velocity followed by an abrupt deceleration at maximum mouth opening position (Figure 7a,b). Conversely, the TP groups (Figure 7c,d) showed a certain degree of cushioning, which could also be consistent with the improvement of the bite-guiding splint.

## 4. Discussion

Currently, magnetic resonance imaging (MRI) has been widely used as a standard diagnostic tool for temporomandibular joint disorders (TMDs) [30]. Although studies have demonstrated a strong correlation between abnormal mandibular movement and TMDs [2], few have adequately focused on evaluating the reliability of mandibular movement function.

This gap is partly due to the lack of suitable devices for recording mandibular movement. Developing a data collection apparatus that moves with the mandible while being lightweight poses significant technical and financial challenges. Another limitation is the absence of consensus on appropriate parameters for assessing mandibular movement. Identifying how to effectively quantify these parameters remains an urgent issue that needs to be addressed.

### 4.1. Technical Advantages

This study proposes a mathematical method to directly solve the motion matrix by using three non-collinear points, with the fourth point used to verify the effectiveness of the method. First, the passive optical tracking system NDI Polaris Spectra was used to collect clinical data. Navigation racks were printed using plastic materials according to the NDI tool design guidelines to minimize interference from the rack weight on mandibular movement. Instead of using physical constraint to match and align coordinate systems [31,32,33], the volunteer received a CT scan while wearing the navigation racks in the current study. Therefore, the possible systematic errors introduced by the registration coordinate system were eliminated. Subsequently, a custom-written quadratic sequence optimization program was compiled in MATLAB (version 2014) based on the principle that rigid bodies do not deform during motion. This program further improved data accuracy at the algorithm level. Finally, the kinetic parameters, including rotational angle, velocity, translational displacement, the position of FHA, etc., could be derived to preliminarily evaluate the bite-guiding splint treatment efficacy.

### 4.2. Clinical Implication

The clinical diagnostic criteria for mandibular movement encompass trajectory reproducibility, movement stability, symmetry (sagittal plane movement deviation), and smoothness (abnormal changes in speed). Existing studies showed a correlation between mandibular movement function and clinical manifestations of joint disease [34], with changes in mandibular movement clinical signs aiding in pathological analysis. In this study, five sets of opening–closing movements from a volunteer were collected for both MIP and TP. The analysis revealed that after wearing the bite-guiding splint, the reproducibility of the trajectories improved, and the movements became more stable. While these changes may be difficult to perceive directly through routine observation, they are clearly reflected in current quantified parameters. This improvement may be due to changes in joint structure, aligning with clinical understanding and consistent with previous findings from our research team [35,36]. This might possibly be used as an important reference for clinical diagnosis or treatment planning, after further investigation provides more information about the reliability of the method and greater convenience.

### 4.3. Significance and Practical Value of FHA Drift

The “hinge axis” hypothesis assumes that during mouth opening, the mandible carries pure rotational movement around a fixed horizontal axis connecting the geometric centers of bilateral condyles. This hypothesis aims to eliminate mandibular translation and simplify analysis. The clinical significance of this hypothesis lies in its ability to provide a method for transferring the occlusal relationship to a mechanical articulator, thereby approximating the mandibular opening movements. However, this approach introduces significant random systematic error, which cannot be repeated or neutralized, leading to an ambiguous understanding of the mandibular movement characteristics. This study calculated the drift of the FHA position during opening–closing movements and refuted the existence of a fixed hinge axis. Instead, the condylar trajectory was calculated more accurately by using motion matrix mapping. The range and stability of the FHA drift before and after wearing the bite-guiding splint differed. These preliminary findings suggest that the proposed method holds potential kinematic value for quantitatively characterizing mandibular movements, though its direct clinical implications remain to be elucidated in future studies with larger sample sizes.

### 4.4. Limitations and Future Prospects

The navigation rack was fixed to the maxilla by using a clamp, and the volunteer followed the instructions of an experienced dentist to complete various mandibular movements for a relatively long time, which may cause slight initial displacement; this technical flaw cannot be ignored. Future research plans will adopt lightweight designs to reduce device vibration and introduce modular and detachable structures to better meet the individual needs of different patients. Currently, we are trying to adopt an individually fabricated titanium navigation rack, which is significantly smaller, lighter, and with higher rigidity to ensure greater precision (Figure S3, Supplementary Materials S5).

As a preliminary proof-of-concept, a single volunteer case was conducted to validate the feasibility of the proposed approach and processing workflow. While the results demonstrated promising consistency with clinical observations, these exploratory findings cannot represent universal patterns or be used to guide clinical decisions at the current stage. To carry this workflow from a proof-of-concept to a clinically viable tool, larger sample sizes are warranted to establish robust databases and diagnostic sensitivity.

The current study lacked extra advanced optical tracking instrumentation for external validation. Although the optimized processing of our internal data is encouraging, the absence of external comparison remains a limitation. Further external validation and phantom validation from another jaw-tracking system with an independent gold standard should be performed in the future to verify the entire data acquisition process.

## 5. Conclusions

This study proposed a mathematical method to decompose opening–closing movement into translational and rotational components. The collected data were optimized through a customized program, and the effectiveness of the method was demonstrated. By quantifying kinematic parameters such as translation increment and rotational angular velocity increment, we analyzed opening–closing movement in the volunteer with and without a bite-guiding splint in different initial jaw positions. Furthermore, our study analyzed changes in the spatial position of the FHA during opening movement to elucidate how mandibular movement occurs, providing a deeper understanding of mandibular movement. The clinical significance of this study lies in its method for recording and analyzing mandibular movement, which surpasses traditional “hinge axis”-based techniques in both information fidelity and clinical usability. Aberrations in TMJ structure can compromise mandibular movement stability, inducing characteristic trajectory alterations. These sensitive kinematic features hold potential for aiding clinical assessment of joint health status and merit further clinical investigation.

## Figures and Tables

**Table 1:** The comparison of maximum differences between “Captured trajectory (CaT)—Derived trajectory (DeT)” and “Captured trajectory (CaT)—Optimized trajectory (OpT)”.

Components	CaT—DeT	CaT—OpT
x	0.2856 mm	0.0569 mm
y	0.6453 mm	0.1904 mm
z	0.0953 mm	0.0379 mm

## Data Availability

The directly recorded data and customized codes used in our study can be obtained from the corresponding author upon reasonable request.

## Supplementary Materials

The following supporting information can be downloaded at: https://www.mdpi.com/article/10.3390/bioengineering13060645/s1. Supplementary Material S1. A proof that four points in the samle plane are insufficient for solving corresponding homogeneous transformation matrix; Supplementary Material S2. A proof that rigid-body motion can be decomposed into translation and rotation motion; Supplementary Material S3. Figure S1 The trajectories of four markers in 3D space for MIP (black) and TP (blue); Supplementary Material S4. Figure S2 The angular velocities of the volunteer at the initial position (MIP) and after wearing bite-splint jaw position (TP) were homogenized to different degrees; Supplementary Material S5. Figure S3 Navigation rack improvement trail: (a) Smaller titanium rack was designed and fabricated using the individualized dental cast data; (b) Higher material strength entitled the lighter weight and smaller size, which reduced the hamper to the mandible movement when wearing it.

## Abbreviations

The following abbreviations are used in this manuscript:

TMJ	Temporomandibular joint
TMD	Temporomandibular joint disorder
MIP	Maximum intercuspal position
TP	Therapeutic position
FHA	Finite helical axis
MRI	Magnetic resonance imaging
