# Supplementary material for: An Accurate Kinematic Analysis with Clinical Convenience for Decomposing Mandibular Movement into Translational and Rotational Components: A Preliminary Proof-of-Concept Study
Source: Bioengineering (Basel). 2026 May 29;13(6):645. doi: 10.3390/bioengineering13060645 (PMC13295940; doi:10.3390/bioengineering13060645)
Supplement: Supplementary file 1 [file bioengineering-13-00645-s001.zip › bioengineering-4305523-supplementary.pdf]

Supplementary Materials for

An Accurate Kinematic Analysis with clinical convenience for Decomposing Mandibular Movement into Translational and Rotational Components: A preliminary Proof-of-Concept Study

Youyi He [1], Baotian Zhu [1], Haolin Li [2], Deqiang Yin [1,*] and Yang Liu [2,**]

[1] College of Aerospace Engineering, Chongqing University, Chongqing, China.

[2] State Key Laboratory of Oral Disease, National Clinical Research Center for Oral Disease, West China Hospital of Stomatology, Sichuan University, Chengdu, China.

[*] Corresponding author at: No.174 Shazhengjie, Shapingba district, Chongqing, 400044, China. Email address: deqiang.yin@cqu.edu.cn (D.Yin)

[**] Corresponding author at: No.24, Yihuan Road, Wuhou District Chengdu, 610065, China. Email address: liu@scu.edu.cn (Y.Liu)

Supplementary Material S1.

A proof that four points in the same plane are insufficient for solving corresponding homogeneous transformation matrix.

Suppose that: there are four points on the same plane: $A_1: (x_1, y_1, z_1\ )$, $A_2: (x_2, y_2, z_2)$, $A_3: (x_3, y_3, z_3)$, $A_4: (x_4, y_4, z_4)$.

The homogeneous coordinate matrix:

$$\begin{pmatrix} x_1 & x_2 & x_3 & x_4 \\ y_1 & y_2 & y_3 & y_4 \\ z_1 & z_2 & z_3 & z_4 \\ 1 & 1 & 1 & 1 \end{pmatrix} \tag{S1}$$

The plane of these four points, denoted: $\mathrm{a} * x + \mathrm{b} * y + \mathrm{c} * z + \mathrm{d} = 0$. This represents the most general case. When $c \neq 0$, we have $z = -\mathrm{d/c} - \mathrm{a} * x/\mathrm{c} - \mathrm{b} * y/\mathrm{c}$. By performing elementary row operations on the homogeneous coordinate matrix mentioned above, it can be found that:

$$\begin{pmatrix} x_1 & x_2 & x_3 & x_4 \\ y_1 & y_2 & y_3 & y_4 \\ z_1 & z_2 & z_3 & z_4 \\ 1 & 1 & 1 & 1 \end{pmatrix} \xrightarrow[\mathrm{r_2*b/c}+r_3]{\substack{\mathrm{r_4*d/c}+r_3 \\ \mathrm{r_1*a/c}+r_3}} \begin{pmatrix} x_1 & x_2 & x_3 & x_4 \\ y_1 & y_2 & y_3 & y_4 \\ 0 & 0 & 0 & 0 \\ 1 & 1 & 1 & 1 \end{pmatrix} \tag{S2}$$

This demonstrates that a homogeneous coordinate matrix of four coplanar points is singular and thus cannot be used to solve for the corresponding homogeneous transformation matrix. The proof for $c = 0$ is analogous and is omitted hereafter.

Supplementary Material S2.

A proof that rigid - body motion can be decomposed into translation and rotation motion.

The 3D motion of a rigid body can be represented by the homogeneous transformation matrix ***E***:

$$E = \begin{pmatrix} R & T \\ 0 & 1 \end{pmatrix} \tag{S3}$$

Considering a change of coordinate system basis for ***E***, assume the new coordinate system basis is obtained by rotating the original coordinate basis via a rotation matrix ***P*** with translation vector c, where $P = (v_1, v_2, u)^T$. Where u is the rotation axis of ***P***, v_1 and v_2 are orthonormal vectors in the rotation plane, and $P^{-1} = P^T$. Taking four non - coplanar points with coordinates matrix:(a_1, a_2, a_3, a_4) and (b_1, b_2, b_3, b_4). The relationship can be expressed as:

$$\begin{pmatrix} b_1 & b_2 & b_3 & b_4 \\ 1 & 1 & 1 & 1 \end{pmatrix} = E * \begin{pmatrix} a_1 & a_2 & a_3 & a_4 \\ 1 & 1 & 1 & 1 \end{pmatrix} \tag{S4}$$

The four points are not coplanar, so

$$E = \begin{pmatrix} b_1 & b_2 & b_3 & b_4 \\ 1 & 1 & 1 & 1 \end{pmatrix} * \begin{pmatrix} a_1 & a_2 & a_3 & a_4 \\ 1 & 1 & 1 & 1 \end{pmatrix}^{-1} \tag{S5}$$

After the original coordinate system undergoes a rotation transformation by ***P*** and a translation by ***c***, the homogeneous transformation matrix ***F*** in the new coordinate system can be expressed as:

$$F = \begin{pmatrix} P & c \\ 0 & 1 \end{pmatrix} * \begin{pmatrix} R & T \\ 0 & 1 \end{pmatrix} * \begin{pmatrix} P & c \\ 0 & 1 \end{pmatrix}^{-1} = \begin{pmatrix} PRP^T & (I - PRP^T) * c + PT \\ 0 & 1 \end{pmatrix} \tag{S6}$$

Observing the top-left 3×3 matrix PRP^T, ***R*** rotates the elements of $\boldsymbol{P^T}$ in the old

coordinate system. Given $P^T = (v_1, v_2, u)$, with the third element u being the rotation axis. The rotation operation only involves two sets of orthogonal basis vectors with a rotation angle θ, resulting in $RP^T = (v_1 cos\theta + v_2 sin\theta, -v_1 sin\theta + v_2 cos\theta, u)$. The operation can be expressed in matrix multiplication form as follows:

$$RP^T = (v_1, v_2, u)\begin{pmatrix} cos\theta & -sin\theta & 0 \\ sin\theta & cos\theta & 0 \\ 0 & 0 & 1 \end{pmatrix} = P^T\begin{pmatrix} cos\theta & -sin\theta & 0 \\ sin\theta & cos\theta & 0 \\ 0 & 0 & 1 \end{pmatrix}$$

$$P^T = P^{-1}, PRP^T = \begin{pmatrix} cos\theta & -sin\theta & 0 \\ sin\theta & cos\theta & 0 \\ 0 & 0 & 1 \end{pmatrix} \tag{S7}$$

For the matrix $(I - PRP^T) * c + PT$, let $PT = t = \begin{pmatrix} t_x \\ t_y \\ t_z \end{pmatrix}$, and $c = \begin{pmatrix} c_x \\ c_y \\ c_z \end{pmatrix}$. This can be written as:

$$\begin{pmatrix} 1 - cos\theta & sin\theta & 0 \\ -sin\theta & 1 - cos\theta & 0 \\ 0 & 0 & 1 \end{pmatrix}\begin{pmatrix} c_x \\ c_y \\ c_z \end{pmatrix} + \begin{pmatrix} t_x \\ t_y \\ t_z \end{pmatrix} = \begin{pmatrix} 0 \\ 0 \\ k \end{pmatrix} \tag{S8}$$

When the $\theta \neq 2k\pi$, the matrix $\boldsymbol{F}$ can be expressed as:

$$\begin{pmatrix} cos\theta & -sin\theta & 0 & 0 \\ sin\theta & cos\theta & 0 & 0 \\ 0 \quad 0 & & 1 & k \\ 0 \quad 0 & & 0 & 1 \end{pmatrix} \tag{S9}$$

This demonstrates that rigid-body motion can be decomposed into rotational and translational motion.

Supplementary Material S3.

Figure S1

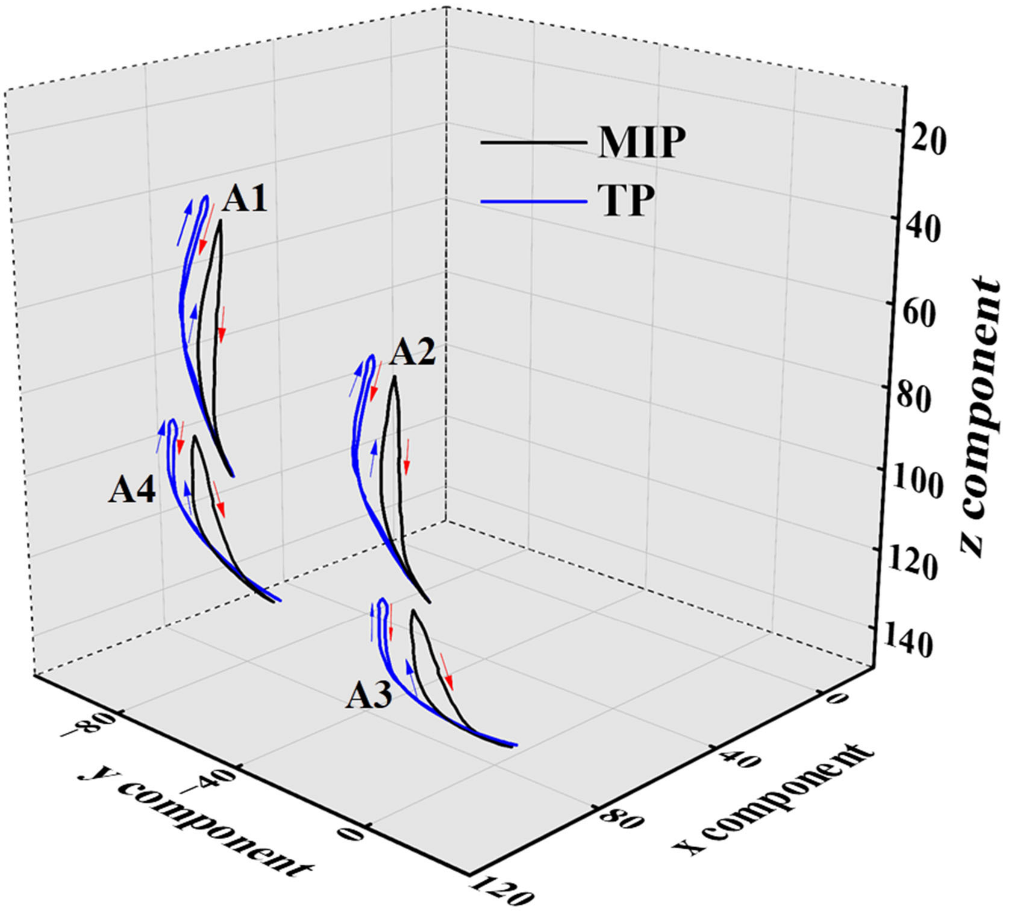

Figure S1 The trajectories of four markers in 3D space for MIP (black) and TP (blue).

Supplementary Material S4.

Figure S2

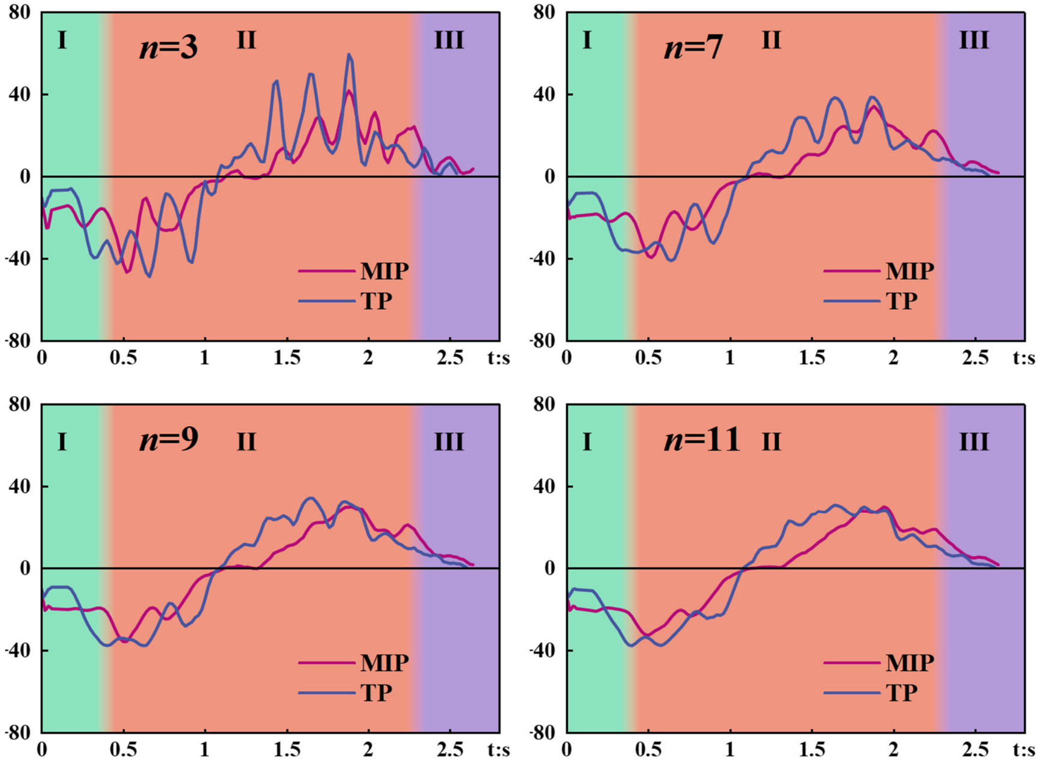

Figure S2 The angular velocities of the volunteer at the initial jaw position (MIP) and after wearing bite-splint jaw position (TP) were homogenized to different degrees.

Supplementary Material S5.

Figure S3

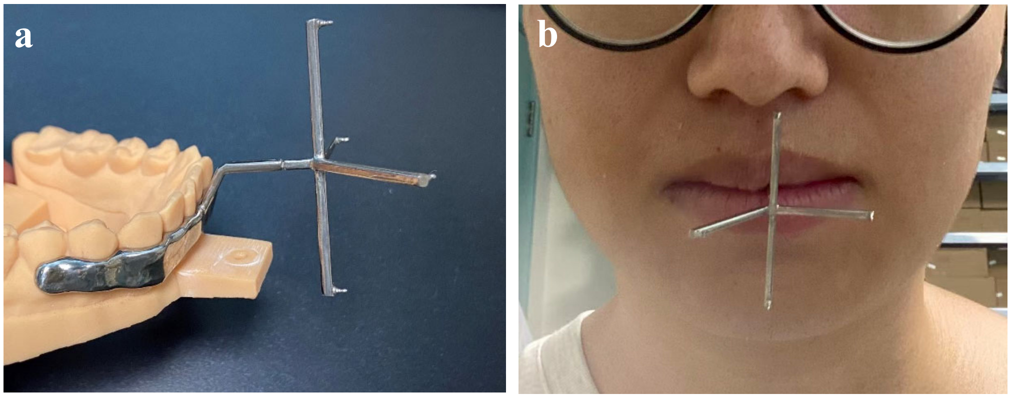

Figure S3 Navigation rack improvement trail: (a) Smaller titanium rack was designed and fabricated using the individualized dental cast data; (b) Higher material strength entitled the lighter weight and smaller size, which reduced the hamper to the mandible movement when wearing it.
